# Comparison of the Micromorphology and Ultrastructure of Pollen Grains of Selected *Rubus idaeus* L. Cultivars Grown in Commercial Plantation

**DOI:** 10.3390/plants9091194

**Published:** 2020-09-12

**Authors:** Mikołaj Kostryco, Mirosława Chwil, Renata Matraszek-Gawron

**Affiliations:** Department of Botany and Plant Physiology, University of Life Sciences in Lublin, Akademicka 15 Street, 20-950 Lublin, Poland; mikolaj.kostryco@up.lublin.pl (M.K.); renata.matraszek@up.lublin.pl (R.M.-G.)

**Keywords:** raspberry, spore, exine sculpture, tectum, columellae, SEM, TEM

## Abstract

The genus *Rubus* is one of the largest taxonomically diverse and complex genera in the family Rosaceae. Morphology of pollen grains (equatorial and polar axes length, shape and size, aperture position, exine sculpture, perforations) is regarded as one of its main diagnostic features for identification of species and varieties. An attempt was made to fill the gap concerning the pollen micromorphology and ultrastructure of *R. idaeus* L. using light, scanning, and electron transmission microscopy. This study is a comparative analysis of micromorphological and ultrastructural traits of pollen from six raspberry cultivars. The pollen grains were classified as small or medium of shape *prolato-spheroids*. The parallel striae in the equatorial view in the exine sculpture were sometimes branched dichotomously in ‘Glen Ample’, ‘Polka’, and ‘Polana’, arcuate in ‘Laszka’ and ‘Pokusa’, or irregularly overlapping in ‘Radziejowa’. The width of exine striae of biennial fruiting cultivars was much larger than in repeated fruiting cultivars. In terms of the increasing number of perforations per unit area of the exine surface, the cultivars were ranked as follows: ‘Pokusa’ < ‘Glen Ample’ < ‘Laszka’ < ‘Polka’ < ‘Polana’ < ‘Radziejowa’. The thickest tectum, the highest and thickest columellae with the largest distances between them, and the thicker foot layer were demonstrated in ‘Glen Ample’. The ectoexine constituted on average ca. 78–90% of the exine thickness. The findings may constitute auxiliary traits i.a. for identification of related taxa, interpretation of phylogenetic relationships, and pollination biology.

## 1. Introduction

*Rubus* is one of the largest genera in the family Rosaceae [1,2,3,4,5,6]. Representatives of the genus have been classified into twelve subgenera: *Anaplobatus, Chamaebatus, Chamarmorus, Comaropsis, Cylactis, Dalibarda, Dalibardastrum, Ildaeobatus, Malachobatus, Micranthobatus, Orobatus,* and *Rubus* [3].

### 1.1. Genus Rubus in the Flora of Poland

In the flora of Poland, *Rubus* plants represent several subgenera: *Idaeobatus*, *Cylactis*, *Chamaerobus*, *Anoplobatus*, and *Rubus*. The subgenus *Idaeobatus* comprises, e.g., the raspberry (*R. idaeus* L.) with red fruits and the Chinese raspberry (*R. xanthocarpus* Bureau. et Franch.) with yellow fruits. The second and third subgenera mentioned include herbaceous plants. One of the species of the subgenus *Cylactis* is the stone bramble (*R. saxatilis* L.) with red fruits. In turn, the genus *Chamaerobus* is represented by the cloudberry (*R. chamaemorus* L.) with yellow fruits, which is under strict species protection. Blackberries are representatives of the last two genera mentioned above. The purple-flowered blackberry (*R. odoratus* L.) belongs to *Anoplobatus*, and the genus *Rubus* comprises, e.g., the four most frequent species in the country, i.e., *R. plicatus* Weihe and Nees, *R. hiatus* Waldst. and Kit., *R. caessius* L., and *R. nessensis* Hall [3,7,8,9,10,11,12,13,14]. In Poland, *R. idaeus* L. is the most common species of the genus *Rubus*. *Rubus* shrubs grow in forests and their margins and in roadside thickets [12,13]. The species has many cultivars used for home-garden cultivation and commercial production [15,16,17,18,19,20,21].

### 1.2. Economic Importance

Given the commercial production of its fruit, *Rubus idaeus* has considerable economic importance in Europe and Poland [22,23,24,25,26,27]. Raspberry fruit is intended either for direct consumption or for processing in many industries: food [20,28,29,30,31], pharmaceutical [20,28,32,33], cosmetic [28,30,33,34,35,36], and nanotechnological [37,38,39,40] industries. Raspberry shrubs are a source of medicinal and cosmetic raw materials, i.e., *Rubi idaeus fructus* [41,42,43,44,45] and *Rubi idaeus folium* [20,46,47,48]. In early spring, abundantly flowering inflorescences provide nectar and pollen reward for pollinating insects, including the honeybee [49,50]. Raspberry nectar and pollen constitute an important ingredient of bee products [51,52].

### 1.3. Morphology of Pollen from the Subfamily Rosoideae

The genus *Rubus* is taxonomically complex and exhibits high morphological diversity. The characterization and infrageneric classification of its species are complicated by frequent processes of hybridization, polyploidy, and agamospermy [53,54,55,56,57,58]. Although some species from the Rosaceae family are stenopalynous plants, e.g., *Rubus* and *Potentilla*, [59,60,61,62], they vary in the structure of pollen grains, which are used as indicators of taxonomic affiliation [60,63,64,65,66].

Pollen grains in plants from the subfamily Rosoideae represent the tricolpate-porate type [67,68,69,70,71,72,73]. The exine of pollen grains in, e.g., *Alchemilla occidentalis, Amelanchier alnifolia, Aruncus sylvester, Chamaerhodos erecta*, and various species of the genus *Rubus* bears perforations [59,63,64,74,75,76].

The following shapes of pollen grains were noted in some species of the genus *Rubus*: perpolate, oblato-spheroides, perprolate, prolate, prolato-spheroides, prolatum, spheroidal, subprolatum, and subspheroidal [65,72,73,74,75,76,77,78,79].

The exine in various species from the subfamily Rosoideae was found to have mainly striate or reticulate sculpture [59,66,77,80,81,82]. The striate sculpture exhibited a varied degree of development of muri. The striae were found to form various patterns: distinctly striate (*Agrimonia grosephala, Rubus gracilis*), delicately reticulate to slightly striate (*Agrimonia strata*, *Rosa canina*), slightly striate or rugulate (*Chamaerhodos erecta*), scabrate to slightly striate (*Amelanchier alnifolia*)*,* coarsely striate (*Aruncus sylvester*), or striate-rugulate (*Fragaria rubicola*) [63,69]. In other taxa, the sculpture was echinate (*Agrimonia eupatoria*), rugulate (*Agrimonia grosephala*), scabrate (*Alchemilla ypsilotoma*), or slightly scabrate (*Alchemilla occidentalis*) [63,69]. In turn, four exine patterns were distinguished in plants of the genus *Rubus*: rugulate, striate, cerebroid, and perforate-reticulate together with 11 subtypes: rugulate-subpsilate, rugulate-striate, rugulate-perforate, striate, striate-perforate, striate-reticulate, cerebroid, cerebroid-perforate, perforate, perforate-reticulate, and reticulate [77].

The striate sculpture in numerous species of this genus exhibited varied arrangement of muri in relation to the corpus: perpendicular (*R. armeniacus, R. gracilis, R. laciniatus, R. pedemontanus, R. fabrimontanus*), parallel (*R. hirtus*)*,* longitudinal *(R. apricus*, *R. divaricatus, R. nessensis*), and meandering (*R. apricus*) [64]. Zhou et al. [83] have reported that the exine sculpture in the subfamily Rosoideae is the most complex of all subfamilies of Rosaceae.

The molecular regulation of sporopollenin biosynthesis determines the proper formation of the sculpture pattern in pollen grains. The exine provides pollen grains with structural, physical, chemical, and biological protection. Its durability and stability are determined by the presence of sporopollenin [84].

Sporopollenin is a mixture of organic biopolymers [85,86]. The presence of sporopollenin in the exine reduces the bioavailability of nutrients contained in pollen by approximately 50% [87]. Damage to the exine induced by the process of grinding grains resulted in an increase in the digestibility and biological activity of nutrients associated with the increased content of polyphenols. These compounds contributed to an 11-fold increase in antioxidant activity, hence the higher potential of pollen for medical, dietary, and cosmetic use [88].

Many reports indicate that the traits of pollen grain morphology, e.g., the length of the equatorial and polar axes, shape and size of grains, position of the aperture, exine sculpture, and structure of perforations, are important standard taxonomic indicators for identification of species and varieties [73,89,90,91,92,93,94,95,96]. These parameters represent the main diagnostic features for classification of many plant species [59,63,70,76,90,92,97,98,99,100,101,102,103]. This is also confirmed by ecological, geographical, and genetic research [73,96,104,105]. There is only fragmentary information in the literature about the morphology of *R. idaeus* pollen grains visualized by bright field microscopy and sparse data on the micromorphology of grains revealed by scanning electron microscopy. There are no scientific reports on the ultrastructure of *R. idaeus* pollen grains. Similarly, there is no information about pollen grains of raspberry cultivars based on the results from light, scanning electron, and electron transmission microscopy. Therefore, the present study is an attempt to extend the knowledge in this field.

The aim of the study was to conduct comparative analyses of the diagnostic micromorphological traits (the length of the polar and equatorial axes, the size and shape of pollen grains, the exine sculpture, and topography: striae, perforations, microstructures). The study was also aimed at description of the ultrastructural features with analysis of the ecto- and endoexine (thickness of the tectum and foot layer, and the height, thickness, and distance between columellae), the intine, and the protoplast in pollen grains of six *Rubus idaeus* L. cultivars: ‘Glen Ample’, ‘Laszka’, ‘Radziejowa’, ‘Pokusa’, ‘Polka’, and ‘Polana’, which are commonly cultivated for commercial purposes in Poland and Europe.

## 2. Results

### 2.1. Micromorphology of Pollen Grains

The anthers of the *R. idaeus* flowers released pollen grains in the bud burst phase. Most of the anthers released pollen on the first and second day of flowering.

#### 2.1.1. Size and Shape of Pollen Grains

The pollen grains of the *R. idaeus* cultivars represent the *tricolporate* type. The length of the polar (P) and equatorial (E) axes in the pollen grains from the biennial fruiting cultivars ranged from 23.8 (‘Radziejowa’) to 28.3 µm (‘Laszka’) and from 21.4 (‘Radziejowa’) to 27 µm (‘Radziejowa’), respectively. In the other group of the cultivars, the mean length of the polar axis in ‘Pokusa’, ‘Polana’, and ‘Polka’ was in the range of 25.4 (‘Polana’)–29.5 µm (‘Polka’). In turn, the length of the equatorial axis ranged from 22.8 (‘Polana’) to 28.6 µm (‘Polka’). Based on the size of the longer axis of the sporomorphs in the *R. idaeus* cultivars, the pollen grains from the first group of cultivars were classified as small and medium, but they were medium in the second group. The mean value of the calculated shape coefficient P/E indicated that the pollen grains of the analyzed cultivars represented *prolato-spheroides* in terms of the shape (P/E=1.01–1.14). Additionally, as indicated by the value of this coefficient in the subsequent years of the study, a part of *prolato-spheroides* shape, pollen grains of *subprolatum* shape (P/E = 1.17) were detected in ‘Laszka’, ‘Polana’, and ‘Polka’ (Table 1).

#### 2.1.2. Exine Sculpture

**Figure 1:**
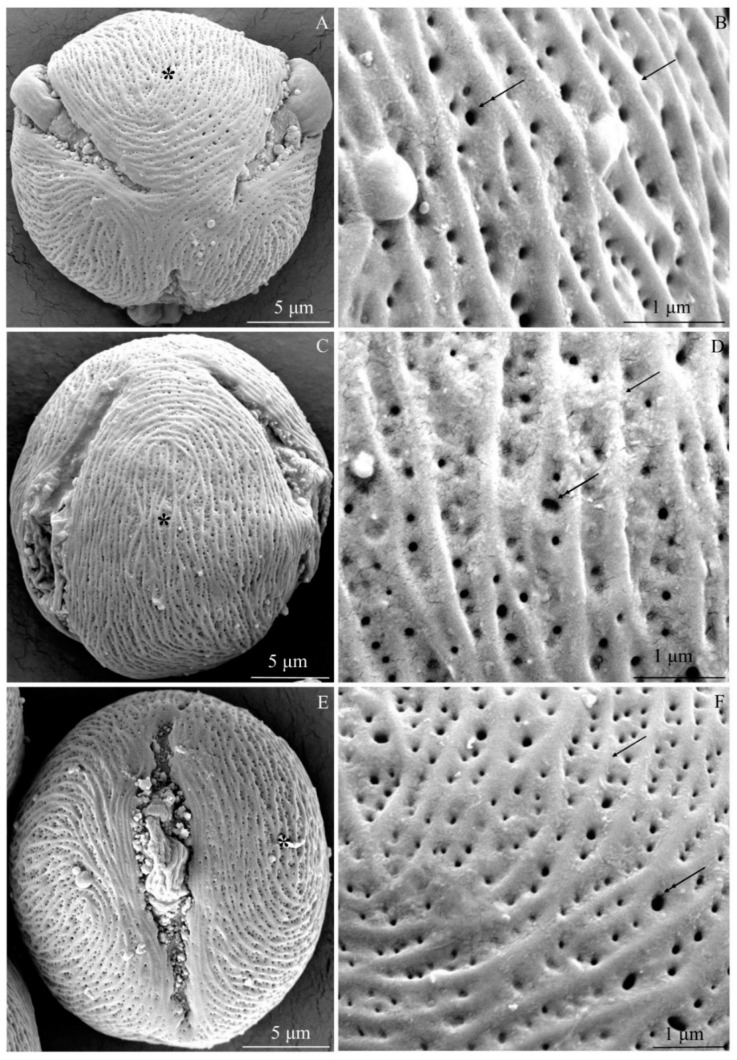
(**A**–**F**). Tricolpate-porate pollen grains (**A**,**C**,**E**) and fragments of exine surface sculpture (**B**, **D**, **E**) in *Rubus idaeus* ‘Glen ample’: (**A**,**B**)—polar view, (**A**) semicircular arrangement of muri close to the apocolpium and parallel or arcuate arrangement between pori, (**B**) exine surface, visible parallel or arcuate arrangement of protuberances (arrow) along the equatorial axis, perforations with different diameters (double-headed arrow) in colpi between muri; (**C**,**D**)—equatorial view, (**C**) muri arranged along the polar axis in the mesocolpium, numerous perforations in colpi, (**D**) perforations aligned in rows, different diameters of perforations (double-headed arrow), varied width of colpi between muri (arrow); (**E**)—colpus and porus in the equatorial plane, visible colpus reaching the distal and proximal poles; (**F**)—varied diameter of perforations (double-headed arrow), visible muri (arrow). (**A**–**F**) - SEM.

**Figure 2:**
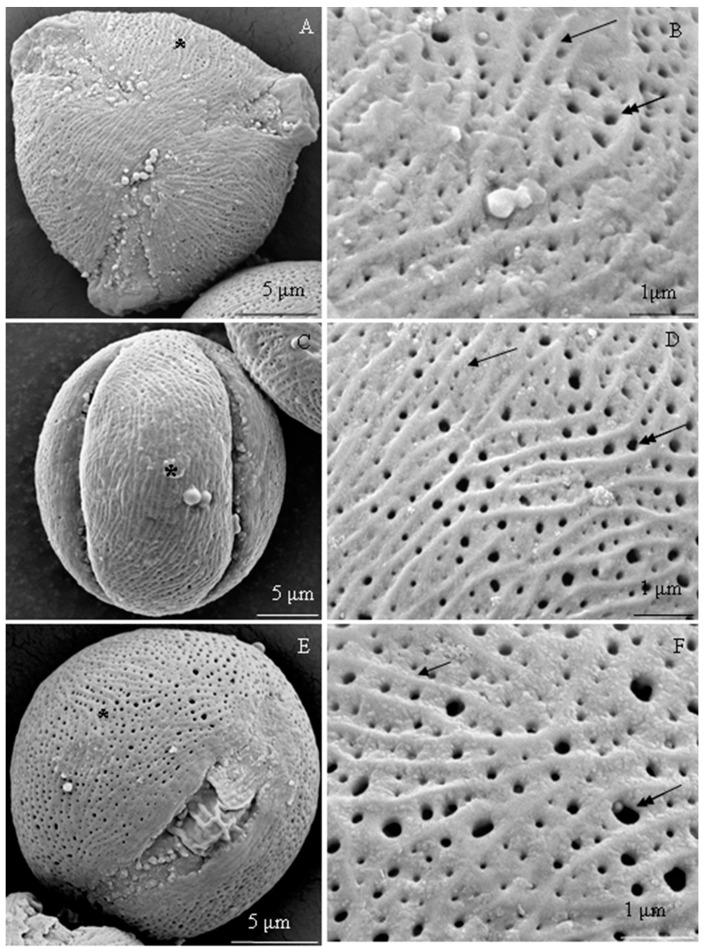
(**A**–**F**). Pollen grains (**A**,**C**,**E**) and fragments of exine surface sculpture (**B**,**D**,**E**) in *Rubus idaeus* ‘Laszka’: (**A**,**B**)—polar view, (**A**) pori visible in colpi, striated surface sculpture and perforations, (**B**)—striated exine surface, different distances between muri (arrow), perforations with different diameters (double-headed arrow) in colpi between muri; (**C**,**D**)—equatorial view, visible muri arranged in parallel and along the polar axis with an undulating or semicircular curve; numerous perforations in colpi, (**D**) muri (arrow) arranged in parallel or in an arcuate curve, dense distribution of perforations (double-headed arrow) in colpi, different diameters of perforations; €—colpus extending to the poles, numerous perforations visible in the exine; (**F**)—perforations (double-headed arrow) arranged in rows in colpi, visible differences in the diameter of perforations. (**A**–**F**) - SEM.

**Figure 3:**
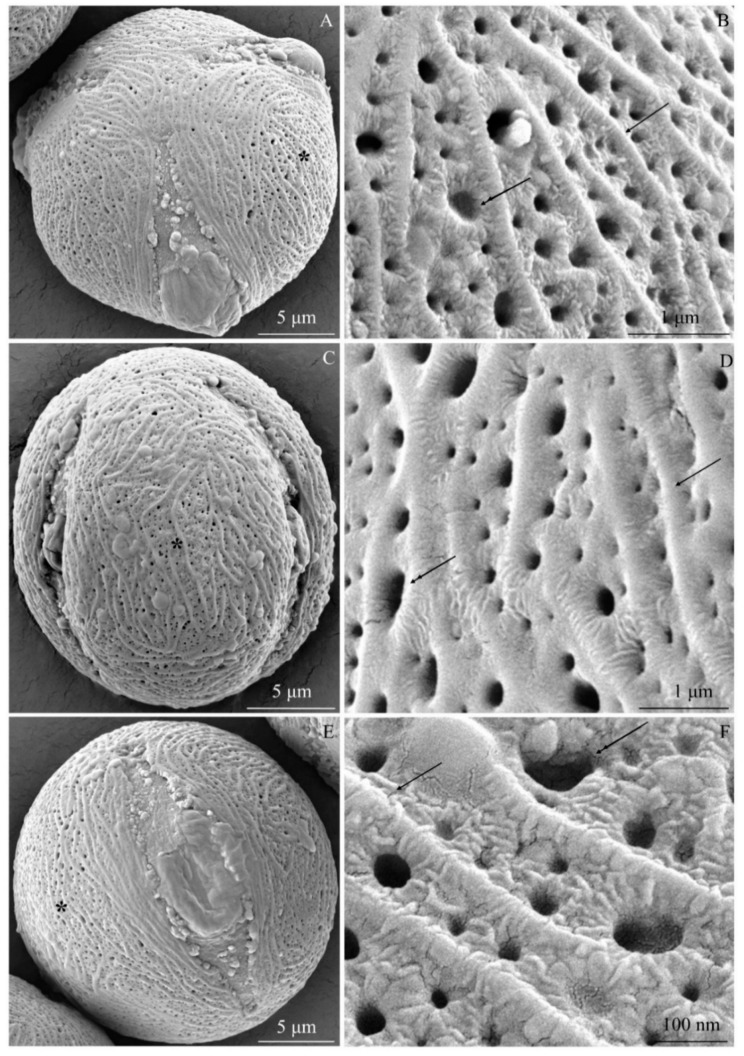
(**A**–**F**). Pollen grains (**A**,**C**,**E**) and fragments of exine surface sculpture (**B**,**D**,**E**) in *Rubus idaeus* ‘Radziejowa’: (**A**,**B**)—polar view, (**A**) muri arranged in parallel along the colpus, with an arcuate bend in the middle part between colpi, sometimes branched near the apocolpium, (**B**) distinctly visible muri (arrow), different width of colpi and diameter of perforations (double-headed arrow), visible striation arranged radially at perforations and perpendicular to muri; (**C**,**D**)—equatorial view, (**C**) muri running towards the polar axis with varied arrangement: parallel, arcuate, and overlapping, (**D**)—perforations with different diameters (double-headed arrow), visible radial exine striation around perforations with different heights of protuberances and arranged perpendicular to muri (arrow); (**E**)—colpus reaching the poles with a visible porus, exine with numerous perforations; (**F**)—several rows of perforations (double-headed arrow) in the colpus, visible delicate striation around the perforation and on the surface of muri (arrow). (**A**–**F**) - SEM.

**Figure 4:**
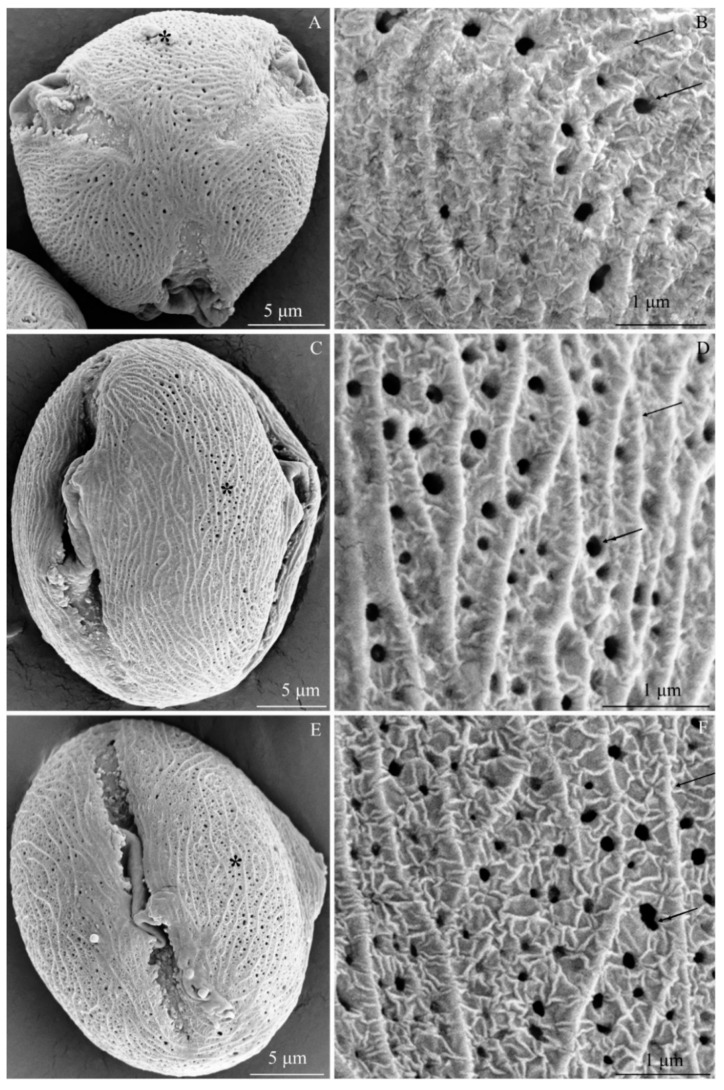
(**A**–**F**). Pollen grains (**A**,**C**,**E**) and fragments of exine surface sculpture (**B**,**D**,**F**) in *Rubus idaeus* ‘Pokusa’: (**A**,**B**)—polar view, (**A**) semicircular muri near the apocolpium and longitudinal, often arcuate, muri between colpi, numerous perforations, (**B**) semicircular arrangement of adjacent muri (arrow), perforations with different diameters (double-headed arrow), visible thin striation with a radial arrangement around the perforation and lateral arrangement on the surface of muri; (**C**,**D**)—equatorial view, (**C**) muri arranged side by side in a parallel, sometimes arcuate or branching arrangement, perforations with different diameters, (**D**) overlapping or parallel muri, fine striae arranged radially around perforations (double-headed arrow) and transversely on the surface of protuberances; (**E**)—equatorial view, colpi reaching the poles, numerous perforations; (**F**)—muri (arrow) in parallel arrangement, sometimes branched, different widths of colpi, fine radially arranged striation (double arrows) around perforations (double-headed arrow), transverse arrangement on the surface of muri, and multidirectional arrangement on the surface of colpi between perforations. (**A**–**F**) - SEM.

**Figure 5:**
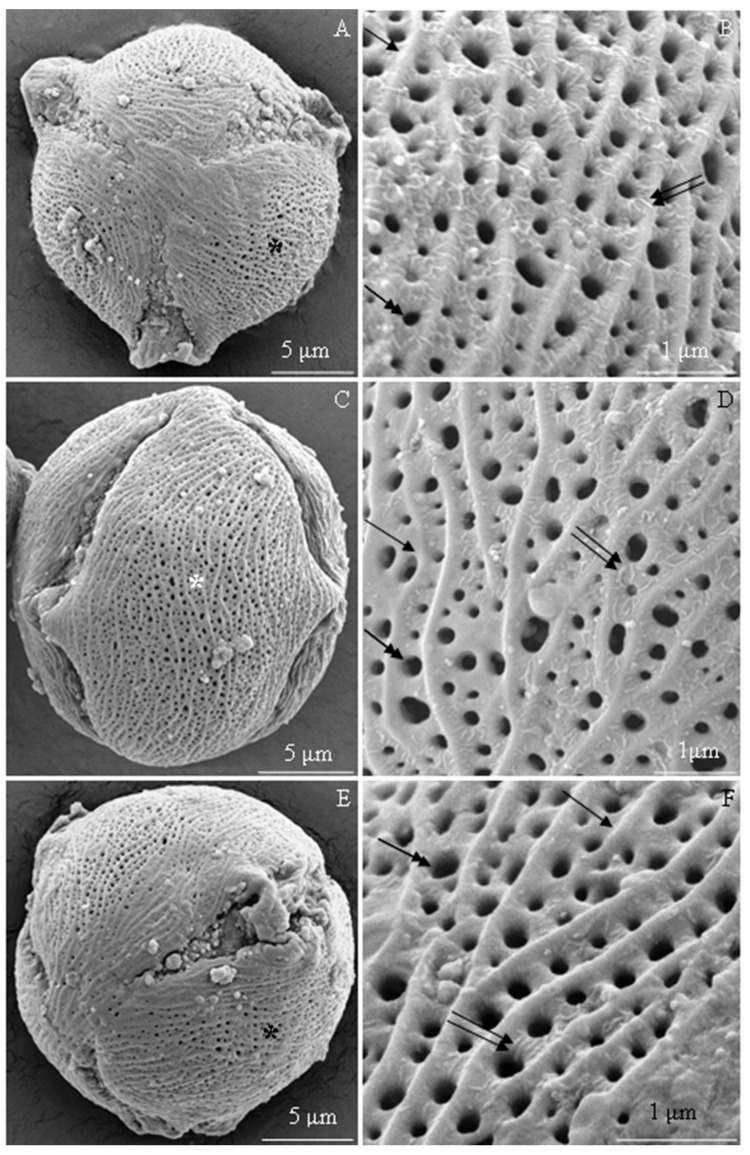
(**A**–**F**). Pollen grains (**A**,**C**,**E**) and fragments of exine surface sculpture (**B**,**D**,**F**) in *Rubus idaeus* ‘Polana’: (**A**,**B**)—polar view, (**A**) parallel arrangement of muri in the apocolpium, arcuate arrangement, or branching between the colpi, (**B**) rows of perforations (double-headed arrow) between muri (arrow), fine striation with characteristic loose radial arrangement of striae around perforations (two arrows) perpendicular to muri; (**C**,**D**)—equatorial view, (**C**) parallel, undulating, and arcuate arrangement of muri along the polar axis, (**D**) striated-perforated exine sculpture, distinct undulating muri, branching in some areas, fine striae in radial arrangement at the perforations (double arrow); (**E**)—equatorial view of a grain, visible colpus with a porus and pollenkitt (arrowhead); (**F**)—rows of perforations (double-headed arrow) in the colpi of parallel muri (arrow) with a semicircular curve, dichotomously branched muri along the polar axis, fine striae around perforations (double arrow) and perpendicular arrangement on the surface of muri. (**A**–**F**) - SEM.

**Figure 6:**
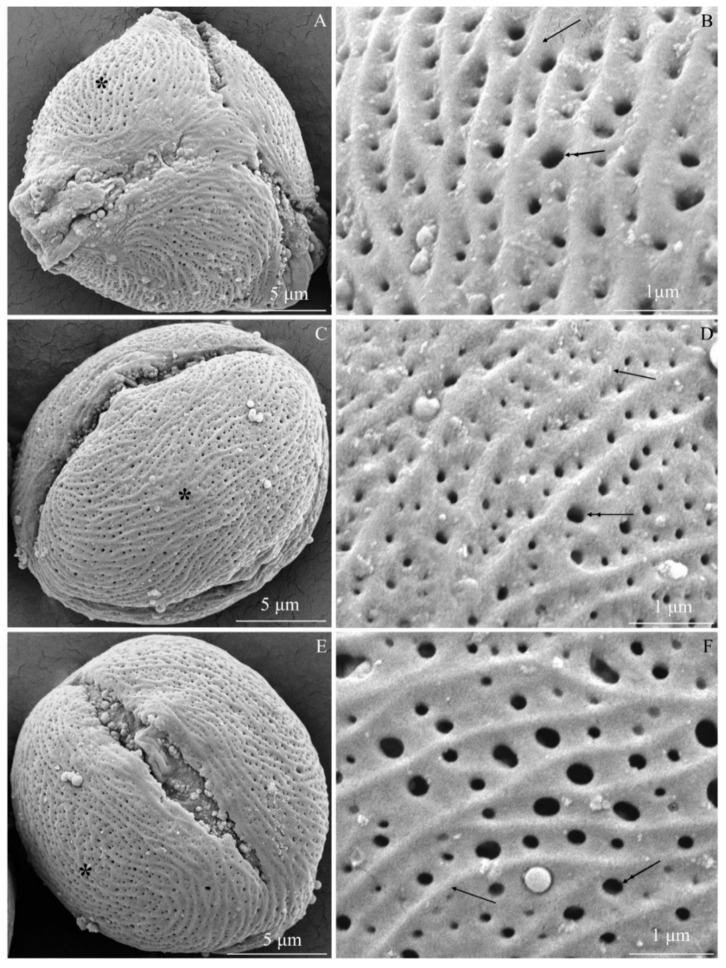
(**A**–**F**). Pollen grains (**A**,**C**,**E**) and fragments of exine surface sculpture (**B**,**D**,**F**) in *Rubus idaeus* ‘Polka’: (**A**,**B**)—polar view, visible delicate outline of arcuate or branched muri in the apocolpium, distinct protuberances between colpi, perforations in colpi, (**B**) dense parallel to semicircular arrangement of muri, varied diameter of perforations arranged in rows between parallel muri; (**C**,**D**)—equatorial view, (**C**) colpi reaching the poles, numerous perforations, parallel arrangement of muri along the polar axis with a semicircular curve, (**D**) parallel arrangement of muri (arrow), varied diameter of perforations between muri; (**E**)—equatorial view of a grain, visible colpus reaching the poles, numerous perforations; (**F**)—parallel and branched muri (arrow), varied arrangement and diameter of perforations (double-headed arrow). (**A**–**F**) - SEM.

The examined *R. idaeus* cultivars had a striated exine with visible perforations (Figure 1A–F, Figure 2A–F, Figure 3A–F, Figure 4A–F, Figure 5A–F, Figure 6A–F). In the polar view, the pollen grains had a triangular outline. There were colpi and pori in the equatorial plane. The colpi were arranged longitudinally. The striae formed a cultivar-specific pattern. On the surface of the *apocolpium*, they were arranged side by side with a delicate arcuate curve in ‘Glen Ample’, ‘Radziejowa’, and ‘Polka’ (Figure 1A, Figure 3A, Figure 6A). They were arranged in parallel in ‘Laszka’ and ‘Polana’ (Figure 2A, Figure 5A) or intertwined in ‘Pokusa’ (Figure 4A) towards the *mesocolpium*.

In the equatorial view, the pollen grains had an elliptical shape (Figure 1C, Figure 2C, Figure 3C, Figure 4C, Figure 5C, Figure 6C). The muri were arranged along the polar axis. On the *mesocolpium* surface, the striae in the exine sculpture in all cultivars were arranged side by side; they were sometimes dichotomously branched in ‘Glen Ample’, ‘Polka’, and ‘Polana’ (Figure 1C,D, Figure 4C,D, Figure 5C,D), arcuate in ‘Laszka’ and ‘Pokusa’ (Figure 2C,D, Figure 6C,D), or irregularly arranged and overlapping in ‘Radziejowa’ (Figure 3C). The colpi in the analyzed pollen grains were long and almost reached the poles (Figure 1E, Figure 2E, Figure 3E, Figure 4E, Figure 5E, Figure 6E).

**Striae.** The width of the striae in the exine varied (Figure 1D, Figure 2D, Figure 3D, Figure 4D, Figure 5D, Figure 6D). The parameter ranged from 248 (‘Radziejowa’) to 417 nm (‘Glen Ample’) in the biennial fruiting cultivars and from 156 (‘Polka’) to 203 nm (‘Polana’) in the other group. The largest and smallest distance between muri in the biennial fruiting cultivars was found in ‘Radziejowa’ (786 nm) and ‘Laszka’ (574 nm), respectively. This parameter of the exine of cultivars with repeated fruiting ranged from 440 nm in ‘Polka’ to 686 nm in ‘Pokusa’. The width of muri in exine of ‘Glen Ample’ was significantly larger than in ‘Laszka’ and ‘Radziejowa’. The distance between muri was significantly lower in ‘Laszka’ than in ‘Glen Ample’ and ‘Radziejowa’. Width of muri in the repeated fruiting cultivars was significantly larger in ‘Polana’ than in ‘Pokusa’ and ‘Polka’, and the distance between muri in ‘Pokusa’ was significantly greater than in ‘Polana’ and ‘Polka’.

The number of striae per 10 µm^2^ of the exine ranged from 8 (‘Glen Ample’) to 11 (‘Laszka’, ‘Radziejowa’) in the biennial fruiting cultivars and from 11 (‘Pokusa’, ‘Polana’) to 13 (‘Polka’) in the repeated fruiting cultivars. The statistical analysis revealed that the number of muri in exine of ‘Glen Ample’ was significantly lower than in ‘Laszka’ (Table 2).

**Perforations.** The perforations in the exine had a spherical or elliptical outline and a varied diameter (Figure 1B,D,E, Figure 2B,D,E, Figure 3B,D,E, Figure 4B,D,E, Figure 5B,D,E, Figure 6B,D,E). These perforations were very small, medium-sized, or large. In the biennial fruiting cultivars, the smallest diameter of the large perforations was found in ‘Glen Ample’ (118–152 nm) and the biggest diameter was exhibited by ‘Laszka’ (249–324 nm). In turn, the smallest and biggest values of this parameter in the cultivars with repeated fruiting were measured in ‘Polka’ (131–173 nm) and ‘Polana’ (194–269 nm), respectively. In the group of the biennial fruiting cultivars, the diameter of perforations in the tectum of ‘Glen Ample’ was significantly lower than in ‘Radziejowa’ and ‘Laszka’. The value of the parameter in the second group cultivars was significantly lower in ‘Polka’ than in ‘Pokusa’ and ‘Polana’. As shown by the comparative analysis of the biennial and repeated fruiting cultivars, the ‘Glen Ample’ cultivar had a comparable diameter of perforations to that in ‘Polka’ and a significantly lower value than in ‘Laszka’, ‘Radziejowa’, ‘Pokusa’, and ‘Polana’ (Table 3). Perforations in the colpi were usually arranged in series and sometimes formed two rows or were scattered irregularly (Figure 1B,D,E, Figure 2B,D,E, Figure 3B,D,E, Figure 4B,D,E, Figure 5B,D,E, Figure 6B,D,E).

The number of perforations in a 2-μm long colpus fragment in the examined cultivars was similar (except in the exine of ‘Pokusa’ pollen) and ranged from 5 (‘Glen Ample’) to 7 (‘Polka’). In turn, there were 39–92 perforations per 10 µm^2^ of the exine surface. Considering the increasing number of perforations per exine unit area, the cultivars were ranked as follows (perforations/10 µm^2^): ‘Pokusa’ (39) < ‘Glen Ample’ (59) < ‘Laszka’ (65) < ‘Polka’ (72) < ‘Polana’ (75) < ‘Radziejowa’ (92). In the group of the biennial fruiting cultivars, the number of perforations in the tectum of ‘Radziejowa’ was significantly higher than the value of this parameter in ‘Glen Ample’ and ‘Laszka’. The value of this parameter in pollen grains of ‘Pokusa’, i.e., a repeated fruiting cultivar, was significantly lower than in ‘Polana’ and ‘Polka’. The comparison of the biennial and repeated fruiting cultivars showed a significantly lower number of perforations per 10 µm^2^ of exine than in the other cultivars (Table 3).

### 2.2. Ultrastructure of Pollen Grains

#### 2.2.1. Pollen Grain Cell Wall 

The transmission electron microscopy observations revealed morphometric differences in the structure of the elements of the ectoexine (tectum, columellae, foot layer) and the endoexine and intine between some of the *R. idaeus* cultivars examined.

**Figure 7:**
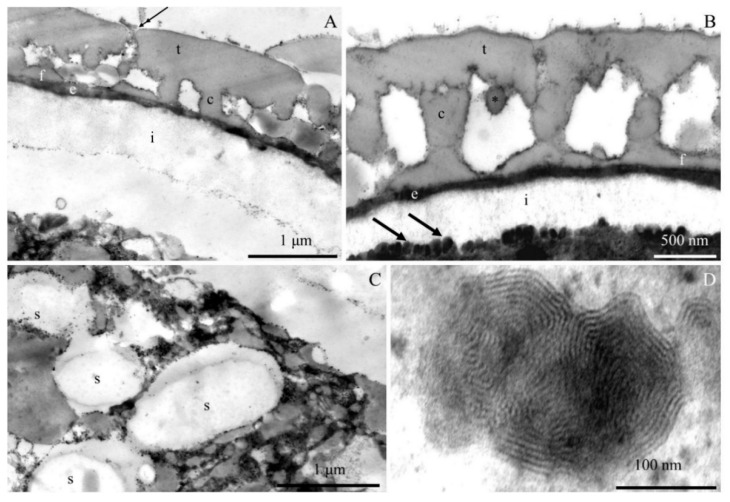
(**A**–**D**). Fragments of pollen grains in *R. idaeus* ‘Glen Ample’: (**A**,**B**)—tectum (t), perforations (double-headed arrow), pollenkitt (asterisk), columellae (c), foot layer (f), endoexine (e), intine (i), numerous electron-dense structures (arrows) indicated by arrows in photo. B; (**C**)—protoplast with electron-dense cytoplasm, starch grains (s) in amyloplasts; (**D**)—characteristic concentric arrangement of electron-dense structures marked with arrows in phot. B. (**A**–**D**) - TEM.

**Figure 8:**
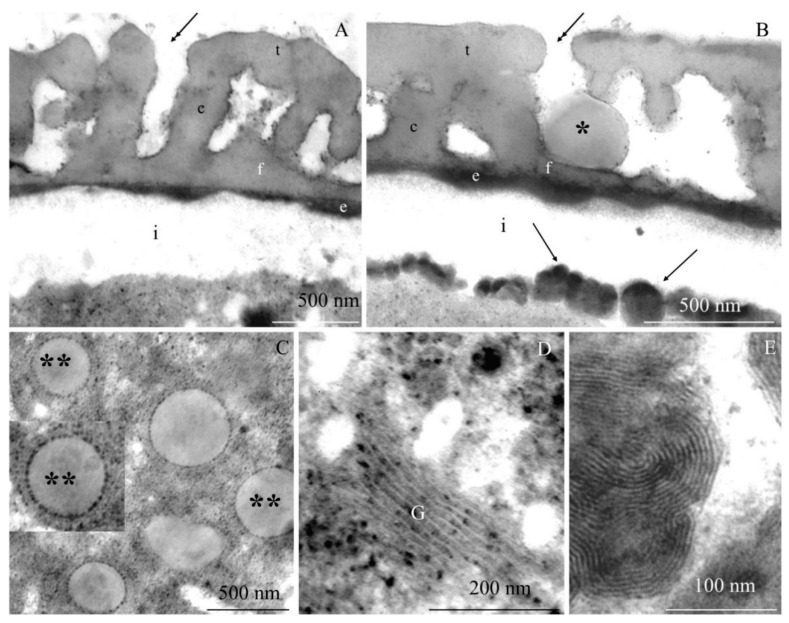
(**A**–**E**). Fragments of pollen grains in *R. idaeus* ‘Laszka’: (**A**,**B**)—perforated tectum (t), visible perforations (double-headed arrow), columellae (c), pollenkitt (asterisk), foot layer (f), endoexine (e), numerous electron-dense structures (arrow) located at the intine (i); (**C**)—electron-dense cytoplasm with numerous plastoglobules (two asterisks); (**D**)—Golgi apparatus (G); (**E**)—concentric lamellar system of electron-dense structures marked with arrows in photo. B. (**A**—**E**) – TEM.

**Figure 9:**
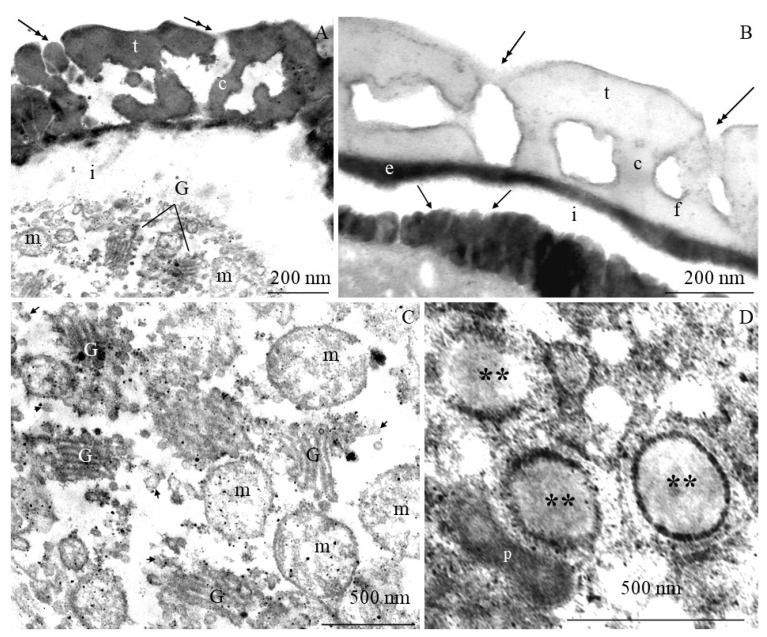
(**A**–**G**). Fragments of pollen grains in *R. idaeus* ‘Radziejowa’: (**A**,**B**)—tectum (t) with perforations (double-headed arrow), columellae (c), foot layer (f), endoexine (e), Golgi apparatus (G) and a mitochondrion (m), e electron-dense structures (arrows) located at the intine (i); (**C**)—numerous mitochondria (m) and Golgi apparatus (G), dictyosomal vesicles (arrowheads); (**D**)—plastoglobules (two asterisks), plastids (p). (**A**–**D**) - TEM.

**Figure 10:**
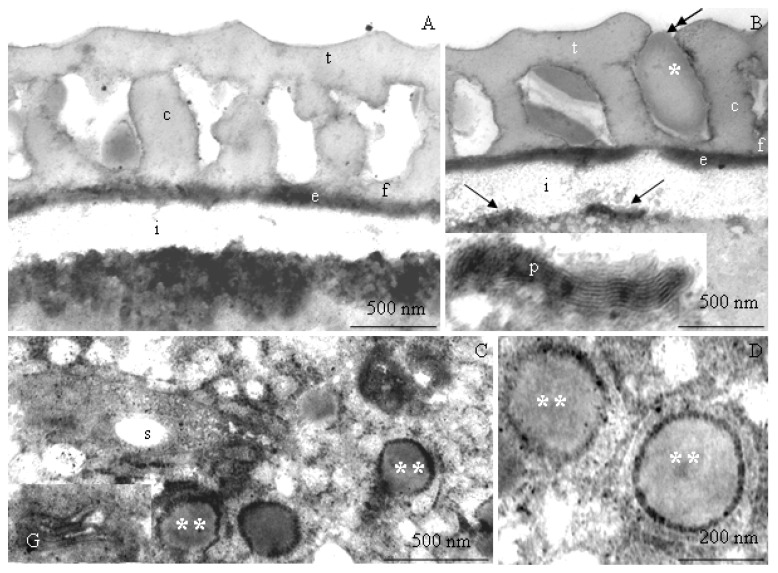
(**A**–**D**). Fragments of pollen grains in *R. idaeus* ‘Pokusa’: (**A**,**B**)—tectum (t), perforation (double-headed arrow), pollenkitt (asterisk), columellae (c) and a foot layer (f), endoexine (e), intine (i) adjacent to numerous electron-dense structures (arrows) in the protoplast; (**C**)—protoplast, visible plastoglobules (two asterisks), mitochondrion (m), starch grain (s) in the amyloplast, Golgi apparatus (G); (**D**)—dense protoplast with plastoglobules (two asterisks), (**A**–**D**) - TEM.

**Figure 11:**
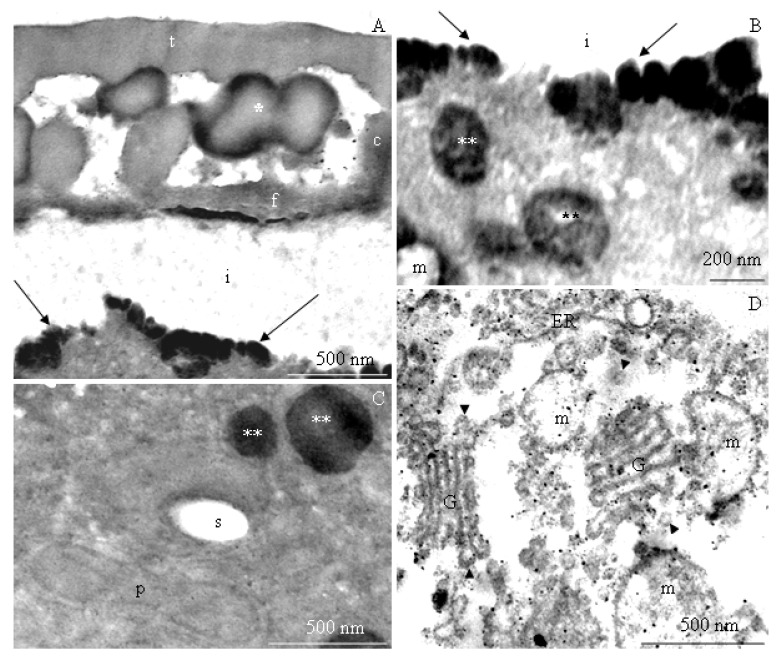
(**A**–**D**). Fragments of pollen grains in *R. idaeus* ‘Polana’: (**A**)—tectum (t), pollenkitt (asterisk), columellae (c), foot layer (f), electron-dense structures in the protoplast (arrows) located at the intine (i); (**B**)—intine (i), numerous electron-dense structures (arrows), plastoglobules (two arrows), mitochondrion (m), elongated plastid (p) with a visible thylakoid system; (**C**)—starch grain (s) in the amyloplast, plastoglobules (two asteriks), plastid (p); (**D**)—Golgi apparatus (G), dictyosomal vesicles (arrowheads), mitochondrion (m), endoplasmic reticulum (ER). (**A**–**D**) - TEM.

**Figure 12:**
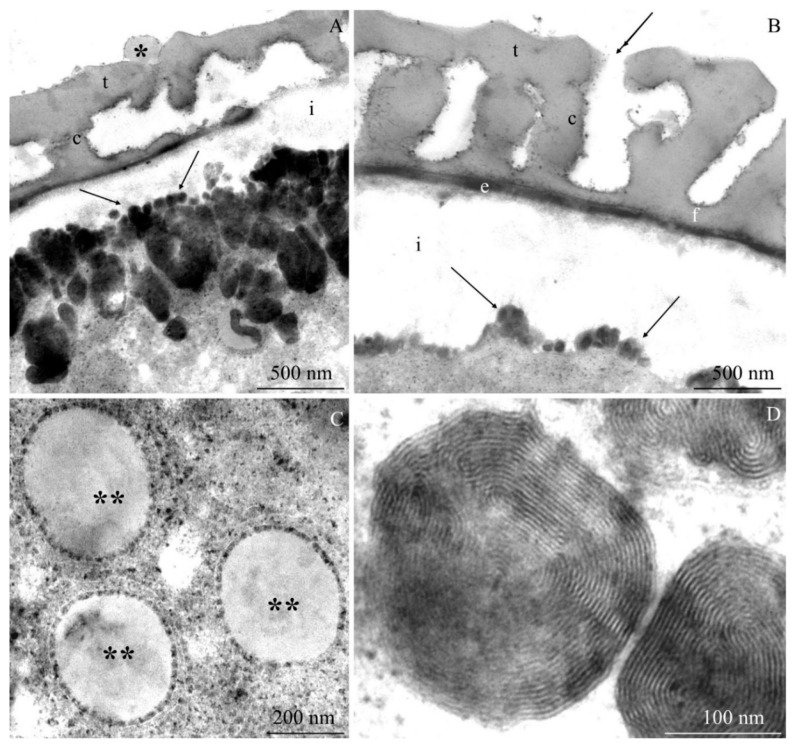
(**A**–**D**). Fragments of pollen grains in *R. idaeus* ‘Polka’: (**A**,**B**)—tectum (t), pollenkitt (asteriks) (phot. A), columellae (c), foot layer (f), endoexine (e), electron-dense structures (arrow) near the intine (i); (**C**)—electron-dense protoplast, plastoglobules (two asterisks); (**D**)—electron-dense structures with a regular concentric arrangement. (**A**–**D**) - TEM.

**Tectum.** A perforated tectum was observed in the exine of the cell wall of pollen grains in the biennial fruiting cultivars (Figure 7A, Figure 8A,B, Figure 9A) and in the repeated fruiting cultivars (Figure 10B, Figure 12B). The thickness of this layer in both groups was in the range of 209 (‘Laszka’)–431 nm (‘Glen Ample’) and 228 (‘Pokusa’)–308 nm (‘Polana’), respectively. In the group of the biennial fruiting cultivars, pollen grains of ‘Glen Ample’ was characterized by a significantly higher value of the thickness of the tectum in comparison with ‘Laszka’ and ‘Radziejowa’, whereas no significant differences in this parameter were found in the group of repeated fruiting cultivars. The comparative analysis between the biennial and repeated fruiting cultivars demonstrated that the ‘Glen Ample’ exine had a significantly higher thickness of the tectum than the other cultivars, with statistically confirmed differences between the ‘Polana’ and ‘Laszka’. The thickness of the tectum was significantly higher in ‘Polana’ than in ‘Laszka’ (Table 4). Electron-dense spherical pollenkitt was visible on the surface of the tectum and between the columellae of the middle layer (Figure 7B, Figure 8B, Figure 10B, Figure 11A, Figure 12A).

**Middle layer.** The height of columellae in the ectoexine in the six analyzed raspberry cultivars ranged from 299 (‘Laszka’) to 403 nm (‘Glen Ample’). The thickness of the columellae was in the range from 208 (‘Pokusa’) to 261 nm (‘Polana’). The distance between adjacent columellae in the biennial fruiting cultivars group ranged from 297 (‘Laszka’) to 407 nm (‘Glen Ample’) nm, while in the group of cultivars with repeating fruits from 321 (‘Pokusa’) to 339 nm (‘Polana’). In the group of the biennial fruiting cultivars, ‘Glen Ample’ had a significantly greater distance between the columellae than ‘Laszka’ and ‘Radziejowa’ (Table 4).

**Foot layer.** The foot layer had an irregular outline on the side of columellae and tightly adhered to the endoexine (Figure 7B, Figure 8A, Figure 9B, Figure 10A, Figure 11A, Figure 12F). This band was the thinnest of the three structural ectoexine layers. Its thickness in the wall of pollen grains of the six analyzed cultivars varied. The value of this parameter ranged from 78 (‘Laszka’) to 149 nm (‘Glen Ample’) in the first group of cultivars and from 109 (‘Pokusa’) to 133 nm (‘Polka’) in the other group. As shown by the statistical analysis performed in the group of the biennial fruiting cultivars, ‘Glen Ample’ was characterized by a significantly higher value of the thickness of the foot layer in comparison with ‘Radziejowa’. In turn, the value of this parameter in ‘Radziejowa’ was significantly higher than in ‘Laszka’. In the group of the repeated fruiting cultivars, the thickness of the foot layer in the ‘Polka’ cultivar was significantly higher than the value of this parameter in ‘Pokusa’ and ‘Polana’. The comparative analysis of the biennial and repeated fruiting cultivars revealed that the thickness of the foot layer in ‘Glen Ample’ and ‘Polka’ was significantly lower than in ‘Radziejowa’, ‘Pokusa’, and ‘Polana’, but the value of the parameter in these three cultivars was significantly higher than that in ‘Laszka’ (Table 4).

The tectum layer, columellae, and foot layer described in the six analyzed cultivars formed an ectoexine with a thickness in the range from 586 nm (‘Laszka’) to 982 nm (‘Glen Ample’). These values accounted for on average 89–92% of the exine thickness (662–1072 nm). As indicated by the statistical analysis performed in the group of the biennial fruiting cultivars, ‘Glen Ample’ exhibited a significantly higher value of the ectoexine and exine thickness than ‘Laszka’ and ‘Radziejowa’. In turn, in the group of the repeated fruiting cultivars, ‘Pokusa’ had a significantly lower exine thickness value than ‘Polana’ and ‘Polka’. The comparative analysis of both cultivar groups revealed a significantly higher value of the ectoexine and exine thickness in ‘Glen Ample’ than that in the other cultivars. ‘Laszka’, ‘Radziejowa’, and ‘Pokusa’ were characterized by significantly lower ectoexine thickness than ‘Polana’ and ‘Polka’. Furthermore, the ‘Polana’ and ‘Polka’ cultivars had a significantly higher value of exine thickness than ‘Laszka’ and ‘Radziejowa’, and the exine thickness in ‘Polka’ was higher than in ‘Pokusa’. Simultaneously, the endoexine thickness in ‘Polka’ was significantly higher than in the other cultivars (Table 5).

**Endoexine.** The endoexine layer in the pollen grains of the studied cultivars ranged from 76 nm ‘Laszka’ to 108 nm ‘Polka’. The ultrastructural study of the cross-sections of the pollen grains of the analyzed cultivars showed that the layer had a regular outline. This electron dense layer was darker than the lighter structural elements of the ectoexine and the unstained intine (Figure 7A,B, Figure 8A,B, Figure 9B, Figure 10A, Figure 12B). The increasing thickness of the exine in the pollen grains ranked the cultivars as follows: ‘Laszka’ < ‘Radziejowa’ < ‘Pokusa’ < ‘Polka’ < ‘Polana’ < ‘Glen Ample’. As indicated by the statistical analysis performed in the group of the biennial fruiting cultivars, ‘Glen Ample’ exhibited a significantly higher value of the ectoexine and exine thickness than ‘Laszka’ and ‘Radziejowa’. In turn, in the group of the repeated fruiting cultivars, ‘Pokusa’ had a significantly lower exine thickness value than ‘Polana’ and ‘Polka’. The comparative analysis of both cultivar groups revealed a significantly higher value of the ectoexine and exine thickness in ‘Glen Ample’ than that in the other cultivars. ‘Laszka’, ‘Radziejowa’, and ‘Pokusa’ were characterized by significantly lower ectoexine thickness than ‘Polana’ and ‘Polka’. Furthermore, the ‘Polana’ and ‘Polka’ had a significantly higher value of exine thickness than ‘Laszka’ and ‘Radziejowa’, and the exine thickness in ‘Polka’ was higher than in ‘Pokusa’. Simultaneously, the endoexine thickness in ‘Polka’ was significantly higher than in the other cultivars (Table 5).

**Intine.** The thickness of the intine layer in the pollen grains of the studied cultivars varied. This layer in the biennial fruiting cultivars was the thinnest in ‘Laszka’ (317 nm) and the thickest in ‘Glen Ample’ (498 nm). In turn, in the group of repeated fruiting cultivars, the thickness of this layer ranged from 229 nm (‘Polka’) to 541 nm (‘Polana’). The thickness of the intine layer accounted for 21–38% of the thickness of pollen grains cells (979–1570 nm). In the group of the biennial fruiting cultivars, pollen grains of ‘Glen Ample’ exhibited a significantly higher value of intine and cell wall thickness in comparison with ‘Radziejowa’ and ‘Laszka’. In the repeated fruiting cultivars, the intine and cell wall thickness was significantly higher than these parameters in the ‘Pokusa’ and ‘Polka’. The comparative analysis of the biennial and repeated fruiting cultivars revealed a significantly higher value of the intine and cell wall thickness in ‘Glen Ample’ and ‘Polana’ in comparison with the other cultivars. The intine thickness in ‘Radziejowa’ and ‘Laszka’ was significantly higher than in ‘Pokusa’ and ‘Polka’ (Table 5).

#### 2.2.2. Protoplast

The electron-dense cytoplasm adhering to the intine of the pollen grains of the *R. idaeus* cultivars exhibited numerous dark stained structures (Figure 7B, Figure 8B, Figure 9B, Figure 10B, Figure 11A,B, Figure 12A,B). Their regular layered structure showed the presence of plastids (Figure 7D, Figure 8E, Figure 10B, Figure 12D). Amyloplasts contained starch granules (Figure 7C, Figure 10C, Figure 11C). The protoplast contained numerous plastoglobules (Figure 8C, Figure 9D, Figure 10C,D, Figure 11B,C, Figure 12C), mitochondria (Figure 10A,C, Figure 11B,D), Golgi apparatus (Figure 8D, Figure 9A,C, Figure 10C, Figure 11D), amorphic plastids (Figure 10B, Figure 11C), and endoplasmatic reticulum (Figure 11D).

## 3. Discussion

### 3.1. Micromorphology of Pollen Grains

The morphology study of the pollen grains of the six *R. idaeus* cultivars showed a length of the polar (P) and equatorial (E) axes of P × E: 23.8–29.5 × 21.4–28.6 µm. These values were similar or higher to those described in the literature for *R. idaeus* and other *Rubus* species, e.g., *R. copelandii*, *R. helvellicus*, *R. saxatilis, R. acuminatissimus*, *R. apricus*, *R. chrysogaeus*, *R. clementis*, *R. diclinis*, *R. ellipticus*, *R. ferdinandi-muelleri*, *R. fraxinifolius*, *R. gracilis*, *R. megacarpus*, *R. montis-wilhelmi*, *R. niveus*, *R. novoguineensis*, *R. papuanus*, *R. royenii*, and *R. trigonus* [60,64,73,74,75]. The values were lower than those reported for e.g., *R. acanthodes*, *R. alpestris*, *R. archboldianus, R. buergeri*, *R. calycinus*, *R. chamaemorus* L., *R. lainzii*, *R. lorentzianus*, *R. macgregrii*, *R. moluccanus*, *R. pectinellus*, *R. praecox*., and *R. seebergensis* [60,64,73,75,78,98,106].

**Size of grains**. The pollen grains in the analyzed *R. idaeus* cultivars were classified as small or medium, as indicated by the length of the longer axis of the sporomorphs. A similar size of pollen grains has been described in other species of the genus *Rubus*, e.g., in *R. acuminatissimus*, *R. chrysogaeus*, *R. clementis*, *R. diclinis, R. ellipticus*, *R. ferdinandi-muelleri*, *R. fraxinifolius*, *R. megacarpus*, *R. montis-wilhelmi*, *R. niveus*, *R. novoguineensis*, *R. papuanus*, *R. pectinellus*, *R. royenii* var. *ikilimbu*, and *R. trigonus*. Medium-sized pollen grains were detected in e.g., *R. copelandii*, *R. chamaemorus*, *R. calycinus*, *R. alpestris*, and *R. archboldianus* [74,75,98]. The variation in the pollen grain size may result from the pollination and habitat conditions, whereas the pollen grain transport conditions have been found to play a negligible role in the evolution of the pollen size in species pollinated by bees and birds. The change in the grain size has been shown to influence pollen germination, pollen tube growth, and fertilization [107,108]. The pollen grain size may be modified by the content of nitrogen and phosphorus in the soil. Higher concentrations of these elements have been shown to increase the size, yield, and germinability of grains and enrich their chemical composition. It was also found to have a positive effect on the number of produced seeds [109]. In turn, it has been demonstrated that defoliation of shoots reduces pollen grain size, inhibits the growth of pollen tubes, and reduces the number of seeds [110]. Pollen morphology is affected by climate change. There is a documented relationship between reduced water availability and an increased grain size, which is regarded as an indicator of long-term climate change [111].

Drought stress during flowering of taxa from the family Rosaceae was reported to reduce the amount of produced pollen, increase the pollen grain size, and slightly change their shape. In such conditions, the pollen grains of *Rubus* and *Rosa* plants were elongated and larger. Such features enhance the chances of survival and increase pollen germination rates [112].

**Shape of pollen grains.** The pollen grains in the analyzed cultivars had the shape of *prolato-spheroides*; or occasionally *subprolatum.* The first type of shape was also described in *R. chrysoageus*, *R. macgregrii*, *R. moluccanus* var. *trilobus*, *R. montis-wilhelmi*, *R. novoguineensis*, *R. papuanus, R. royenii,* and *R. trigonus* [75]. *Subprolatum* and *spheroides* types were identified frequently in *Rubus* plants, whereas the *oblato-spheroides* shape was rarely observed [79]. The shape and brightness of grains have been used as descriptors in the computer classification of pollen based on their morphology and ornamentation. The size and shape of grains are palynological data used, e.g., for automatic identification of a given taxon, often in combination with sculpture analysis [113,114].

**Exine sculpture.** The pollen grains of the studied cultivars had a striated and perforated exine sculpture. Similar striated arrangement has been described in some species of the genus *Rubus*: *R. montanus, R. mollis*, and *R. spiribillei* [73]. Perforated sculpture was found in other species of the subgenera *Chamaebatus, Ideobatus*, and *Micranthobathus* [71,74,75]. In other taxa of this genus, the arrangement of striae formed various surface types: loosely striated (*R. acuminatissimus* and *R. niveus*)*,* densely striated (*R. copelandii*)*,* scabrate-striated (*R. lorentzianus*), and papillate-striated (*R. odoratus*). In turn, the surface in *R. glaucus* was found to be smooth [71,73,75]. The pollen grain sculpture in other *Rubus* taxa exhibited the presence of numerous irregular protuberances forming rugulate (*R. archboldianus*) or scabrate (*R. macgregorii*) surfaces [75]. In turn, in species originating from the Iberian Peninsula, the grain exine was covered with clavate, spherical, or ellipsoidal processes forming a micropapillate or microclavate (*R. genevierii*) pattern [60]. Ghosh and Saha [66] observed reticulate-perforated, granulate, and scabrate sculpture in *R. acuminatus*, *R. ellipticus*, and *R. lineatus*.

**Striae.** The striae in the exine sculpture in the pollen grains of the biennial fruiting cultivars (‘Glen Ample’, ‘Laszka’, and ‘Radziejowa’) were approximately 1.5–2 fold wider than in the repeated fruiting cultivars. The width of the exine striae in ‘Radziejowa’ and ‘Polana’ was similar to that reported in the literature for *R. alpestris* and *R. pedemontanus* (200–250 nm), whereas in the range specified for *R. macgregorii* and *R. nessensis* was 100–380 nm [64,75]. The value of this parameter in the exine of ‘Polka’, ‘Pokusa’, and ‘Laszka’ was similar but ‘Glen Ample’ higher to that of *R. macgregorii* and *R. nessensis* (100–380 nm) but lower than in *R. megacarpus* (600–800 nm) [64,74]. In turn, the width of the striae in the other cultivar group was similar to that reported for *R. acuminatissimus, R. armeniacus, R. laciniatus*, and *R. fabrimontanus* (160–180 nm) [64,75]. There were 8–12 striae per 10 μm of exine surface area in the cultivars examined in this study. The longitudinal axis of adjacent striae was located at a distance of 440–786 nm. As reported by Tomlik-Wyremblewska et al. [64], the width of colpi between striae in the exine of *R. armeniacus, R. hirtus,* and *R. pedemontanus* ranged between 100 and 400 nm.

**Perforations.** There were 39–92 perforations per 10-μm unit area in the exine of pollen grains in the examined cultivars. Their minimum and maximum diameters were in the range of 118–209 nm in ‘Glen Ample’, ‘Pokusa’, and ‘Polka’ and 194–324 nm in ‘Polana’, ‘Laszka’, and ‘Radziejowa’. The former values of the parameter were consistent with or close to the range (50–200 nm) specified for *Rubus* species by Tomlik-Wyremblewska [64], whereas the latter values were close to the upper limit (‘Polana’) or higher (‘Laszka’, ‘Radziejowa’). Exine perforations were also observed in endemic taxa occurring in Polish flora, e.g., *R. chaerophylloides* and *R. posnaniensis* as well as *R. clementis* and *R. megacarpus* representing the subgenus *Micranthobatus* [74,79].

Similar perforations were detected in *R. genevierii* originating from the Iberian Peninsula [60]. They were also observed in the exine of *R. acuminatus, R. chmaemorus, R. ellipticus, R. gracilis,* and *R. lineatus* [66,76,98].

### 3.2. Ultrastructure of Pollen Grains

Using a transmission electron microscope, a detailed comparison of the cell wall of pollen grains of the six studied *R. idaeus* cultivars was carried out for the first time. The tectum in the analyzed cultivars was perforated and had a thickness of 209–431 nm. A perforated tectum was observed in other *Rubus* species as well [66,74,75,115,116,117,118,119,120,121,122].

The columellae present between the tectum and the foot layer in the examined grains were located at a distance of 297–407 nm. They were between 299 and 403 nm high and 208–261 nm thick. Ulrich et al. [123] have reported that the ectoexine is composed of the tectum, infratectum, and foot layer. As shown by Doyle and Endress [124], the infratectum can be granular, intermediate, and columellar. The first form is characteristic for pollen grains of gymnosperms but also occurs in some angiosperm species, mainly as the columellar type [125]. The structural part of the ectoexine, i.e., the foot layer, in the grains of the analyzed cultivars formed a 78–149 nm thick continuous layer. As reported by Ulrich et al. [123], this layer can be either continuous, discontinuous, or absent. The structureless ectoexine in their study formed a continuous layer, and its thickness constituted 8–13% of the thickness of the exine layer. These authors indicate that the endoexine may be continuous or discontinuous, spongy or compact, sometimes present only in apertures, or absent.

The thickness of the exine in the grains was lower in ‘Laszka’, ‘Radziejowa’, ‘Pokusa’, and ‘Polka’ were close to the values, but in the case of ‘Polana’ it was in the range of values determined in other *Rubus* species (0.9–2.4 μm) [64,73,74,78,79,105]. Sporopollenin contributes to high exine stability. It consists of oxidizing polymers of carotenoids, polyunsaturated fatty acids, and phenolic compounds [126]. An analysis of the two structural layers of the exine revealed that the endoexine is more resistant to oxidation than the ectoexine. This is determined primarily by the reduction-resistant sporopollenin accumulated on tuft units originating from plasma membrane glycocalyx rather than the secondary sporopollenin accumulated in the ectoexine [127]. Sporopollenin has a common aliphatic core and various taxon-dependent aromatic side chains [126]. Genes responsible for acyl-CoA-derived tetraketide components are involved in its synthesis [128]. One of them is the LAP3 gene involved in the formation of the normal exine structure and development of pollen. A mutation in this gene was found to lead to formation of a thin exine, disturbances in its sculpture, and metabolic changes in pollen grains [129].

The intine layer in the pollen of the examined cultivars constituted 21–38% of the thickness of the grain cell wall. The intine is usually thicker and composed of two layers at the aperture site [123]. The pectate lyase-like 9 gene (BcPLL9) is involved in intine formation [130]. The structure of intine comprises mainly cellulose, pectins, and callose [123,131,132,133]. The former polysaccharide, which is evenly distributed on the intine surface, is a pollen tube wall progenitor [132,133]. It is formed with the involvement of intine components, e.g., hydrolytic enzymes and hydrophobic proteins maintaining the structural compactness of pollen grains. These substances are necessary for recognition of the receptivity of stigmata and further penetration of the pollen tube through the pistil style [134,135,136].

Numerous plastids, sometimes containing starch granules, were observed in the intine-adhering protoplast of the pollen of the analyzed *R. idaeus* cultivars. Plastoglobules, mitochondria, and Golgi apparatus were visible in the electron-dense cytoplasm. As reported by Pacini et al. [137], this polysaccharide accumulates in pollen grains during development and persists until the last phase of pollen maturation. Next, before anther dehiscence, it is converted into pectin, glucose, fructose, sucrose, and other sugars. The function of starch is associated with the use of pollen by pollinators. Pollen grains with amyloplasts contain lower content of lipid compounds than starchless pollen. Starch also serves a protective function against pollen grain drying [138].

However, the carbohydrate metabolic process varies: the compounds can be utilized directly, transformed into other molecules, or polymerized to form intine, or stored as starch. During pollen development, the amylogenesis cycle may be specific for each species. Starch in pollen grains can be used as a reserve formed through partial hydrolysis. Carbohydrates are utilized as a raw material necessary during pollen development. In the final phase, they accumulate, thus contributing to the ability of pollen to spread spontaneously [136].

## 4. Materials and Methods

The research conducted in 2017, 2018, and 2019 involved six cultivars of *R. idaeus* that are commonly produced in commercial plantations and have been included in the national register. The investigations were conducted on three biennial fruiting cultivars: ‘Glen Ample’, ‘Laszka’, and ‘Radziejowa’ and three repeated fruiting cultivars: ‘Polka’, ‘Polana’, and ‘Pokusa’.

### 4.1. Study Area and Plant Material

The raspberries were grown on a commercial plantation in Blinów II (50°52′57.03″ N; 22°23′2.663″ E), Szastarka commune, Lublin Province, in the south-eastern part of Poland. The shrubs grew at a 0.5 × 2.8 m spacing. Fertilization and protection agents were used in accordance with the raspberry cultivation recommendations. Flowers, i.e., the source of the pollen grains, were collected three times from each cultivar at the initial stage of flowering to determine the morphological, micromorphological, and ultrastructural characteristics of pollen.

### 4.2. Fixation of the Material

Stamens with pollen were collected from the flowers at the initial stage of flowering (the bud burst phase). The anthers were fixed in 4% glutaraldehyde for 6 h at room temperature. Afterwards, the samples were treated with 0.01 M phosphate buffer, pH 7.0, for 48 h at 4 °C.

Different methods were employed depending on the intended use of the sample for (i) light field light microscopy, (ii) scanning electron microscopy, and (iii) transmission electron microscopy.

### 4.3. Light Microscopy (LM)

Pollen for the analyses was sampled three times from 30 randomly flowers of the six cultivars in the consecutive study years. After washing out from anthers with 70% ethyl alcohol, the pollen grains were stained with a 1% safranin solution and sealed in a 50% glycerin solution [139,140]. The microscopic slides were analyzed with a light microscope by measuring the equatorial (E) and polar (P) axes in the light microscope. Comparative observations of the morphology of the pollen grains were carried out using a Nikon Eclipse 90i bright field microscope (Nicon Instruments INC, Tokyo, Japan).

### 4.4. Scanning Electron Microscopy (SEM)

Fixed samples of anthers with pollen grains were dehydrated in a successive 15, 30, 50, 70, 90, and 99.5% acetone series for 15 min at room temperature and twice in anhydrous acetone. Next, the pollen grains were critical point dried in liquid CO_2_ using an Emitech K850 dryer (Quorum Technologies Ltd., Ashford, United Kingdom). Dried grains were transferred onto the microscope stage and sputter-coated with gold using an Emitech K550X device (Quorum Technologies Ltd., Ashford, United Kingdom). Observations of the surface of pollen grain sculpture (striae, perforations, microstriae) and photographic documentation were made using a Tescan Vega II LMU scanning electron microscope (SEM) (Tescan Orsay Holding, Brno, Czech Republic). The microscope was used for analysis of 100–150 pollen grains from each cultivar.

### 4.5. Transmission Electron Microscopy (TEM)

The fixed anthers with pollen grains were contrasted in a 1.5% osmium tetroxide solution for 1.5 h. After rinsing with distilled water, a 0.5% aqueous uranyl acetate solution was applied for 2 h at room temperature. After rinsing with distilled water twice, fragments of anthers and pollen grains were dehydrated for 15 min in a series of ethyl alcohol at subsequent concentrations of 15, 30, 50, 70, 90, 96, and 99.8% and twice in anhydrous ethanol. Dehydrated samples were embedded in Spurr Low Viscosity resin and polymerized at 60 °C for 48 h. The resin-embedded plant material was cut with a glass knife into half-thin sections with a thickness of 1 μm using a Reichert Ultra Cut S microtome; next, the material was contrasted with osmium tetroxide and mounted in Eukit.

In turn, ultrathin sections of the anther with pollen grains with a thickness of 70 nm were stained with an 8% solution of uranyl acetate in 0.5% acetic acid for 40 min. 

After rinsing with distilled water twice for 5 min), Reynolds reagent was applied for 15 min [141]. The sections were rinsed with water again (twice for 5 min) and dried. The structural elements of the ectoexine (tectum, middle, and foot layers), endoexine, intine, and protoplast walls were carried out with the use of the FEI, Tecnai G2 Spirit transmission electron microscope (TEM) (FEI Company, Hillsboro, OR, USA). The comparative analyses of the pollen grain ultrastructure were performed on live images and microphotographs of 3–4 ultrathin sections from each of the 12 blocks in each of the six cultivars. In each block, 2-4 stamen heads were embedded.

### 4.6. Morphometric Measurements

The pollen grains of the *R. idaeus* cultivars were characterized by assessment of (1) the length of the equatorial (E) and (2) polar axes (P) (*n* = 150). Based on these values, (3) the shape index (P/E) was calculated, and (4) the grain shape and (5) size were determined following the classification proposed by Erdman [142] as well as Dybova-Jachowicz and Sadowska [143]. The study of the micromorphology of the exine sculpture in the pollen grains consisted in comparison of (6) the width of striae, and (7) colpi (*n* = 30). The number of (8) striae and (9) perforations per unit area (10 µm^2^) as well as (11) the number of perforations per 2-µm colpus fragment were calculated. The assessment of the cell wall ultrastructure consisted in measurements of the thickness of the following elements: (12) tectum, (13) middle layer, (14) foot layer, (15) ectoexine, (16) endoexine, (17) exine, (18) intine, and (19) cell wall. Additionally, the thickness (20) and height (21) of columellae and (21) the distance between adjacent columellae (*n* = 30) were measured. The morphometric measurements were carried out using Nikon NIS-Elements version 3.0 Advance Research microscopic image analysis software.

### 4.7. Statistical Analysis of Results

The mean results of the morphometric measurements of the micromorphological parameters of the exine sculpture and ultrastructure of pollen grains as well as the standard deviation were calculated using the Microsoft Excel 2013 program. The significance of differences between the examined pollen characteristics was statistically analyzed using the statistical software Statistica 6.0. The differences between the selected traits were evaluated using one-way ANOVA analysis of variance. Statistical inference was carried out at the significance level α < 0.05.

## 5. Conclusions

The pollen grains of the six *R. idaeus* cultivars studied were classified as small or medium, and their shape was classified as *prolato-spheroides* or *subprolatum*. In the striated grain sculpture, adjacent striae in the equatorial position were sometimes dichotomously branched in ‘Glen Ample’, ‘Polka’, and ‘Polana’, arcuate in ‘Laszka’ and ‘Pokusa’, and irregularly arranged and overlapping in ‘Radziejowa’. In comparison with the pollen grains of the biennial fruiting cultivars as compared to the repeated fruiting cultivars, the width of striae and colpi on the exine was 46% and 23%, larger, respectively, and the number of muri per unit area was 14% higher. In terms of the increasing number of perforations per unit area, the cultivars are ranked as follows: ‘Pokusa’ < ‘Glen Ample’ < ‘Laszka’ < ‘Polka’ < ‘Polana’ < ‘Radziejowa’. The ultrastructure analysis demonstrated the thickest tectum in ‘Glen Ample’, the highest and thickest columellae with the largest distance between them in ‘Polka’, and the thickest foot layer in ‘Polana’. The ectoexine in the pollen grains accounted for approximately 89–92% of the thickness of the exine, whereas the intine layer represented 21–38% of the thickness of the grain wall. The present investigations may be helpful indicators for identification of related taxa of the genus *Rubus*.

The micromorphological and ultrastructural traits of the exine in the pollen of the six cultivars of *R. idaeus* documented in the present study may be useful for not only botany, biology, and palynology specialists but also for a wider audience in the field of forestry, horticulture, medicine, apiculture, and sciences related to nutrition and environmental protection.

The parameters of exine sculpture can be used as taxonomic indicators in many fields of science, e.g., in paleoecology and biogeography, archaeological excavations, and interpretation of phylogenetic relationships in the genus *Rubus*. They can also be used in innovative research programs to identify pollen supported by chemotaxonomy and DNA barcoding of plant genomes. These indicators can be useful in practice, e.g., in forensics, biochemistry, biology of the pollination process, assessment of mutualistic relationships, monitoring pollen allergen concentrations, and identification of pollen-based dietary supplement products. They can also be applied in innovative nanotechnology to form microcapsules and natural polymers.

Future research should be focused on micromorphological and ultrastructural analysis as well as microanalysis of pollen in many groups of *Rubus* cultivars in view of their use in cultivation work on, e.g., genetic diversity and resistance to biotic factors and climate change. Investigations should also focus on invasive food processing and nanotechnology as carriers of phytochemicals in pharmacy and cosmetology.

## Figures and Tables

**Table 1:** Length of polar and equatorial axes and the shape index of pollen grains of *R. idaeus* cultivars.

Cultivar	Year	Length of Axis (μm)				P/E	
		Polar Axis (P)		Equatorial Axis (E)			
		Min.–Max.	Mean ± SD	Min.–Max.	Mean ± SD	Min.–Max.	Mean ± SD
**Biennial Fruiting Cultivars**							
‘Glen Ample’	2016	24.40–29.88	27.38 ± 1.31a	24.58-29.54	26.92 ± 1.51a	0.95–1.22	1.02 ± 0.06a
	2017	21.05–27.68	24.57 ± 1.38a	20.60–25.53	23.01 ± 1.40b	1.01–1.13	1.07 ± 0.04a
	2018	24.57–29.71	26.73 ± 1.22a	22.09–26.69	24.77 ± 1.08ab	1.01–1.21	1.08 ± 0.06a
	Mean	-	26.23 ± 1.77A	-	24.90 ± 2.08A	-	1.06 ± 0.06A
‘Laszka’	2016	24.68–30.02	27.55 ± 1.39ab	22.06–25.33	23.49 ± 0.92b	1.02–1.30	1.17 ± 0.08a
	2017	21.30–28.77	24.49 ± 1.99b	19.82–24.84	22.20 ± 1.34b	1.03–1.25	1.10 ± 0.05a
	2018	25.99–28.99	28.29 ± 0.78a	24.15–27.30	25.40 ± 0.87a	1.06–1.20	1.11 ± 0.04a
	Mean	-	26.78 ± 2.20A		23.70 ± 1.69A		1.13 ± 0.07A
‘Radziejowa’	2016	23.00–33.07	26.80 ± 2.40a	23.06–29.95	26.98 ± 1.69a	0.84–1.14	1.01 ± 0.07a
	2017	22.73–26.43	25.13 ± 1.17a	20.96–26.31	23.87 ± 1.61ab	1.00–1.25	1.06 ± 0.06a
	2018	21.29–25.72	23.83 ± 1.23a	18.93–23.97	21.35 ± 1.85b	1.02–1.25	1.12 ± 0.08a
	Mean	-	25.31 ± 1.88A	-	24.01 ± 2.97A	-	1.06 ± 0.08A
**Cultivars With Repeated Fruiting**							
‘Pokusa’	2016	25.20–29.04	27.56 ± 1.06a	25.01–29.46	26.91 ± 1.19a	0.93–1.15	1.03 ± 0.06a
	2017	25.21–31.29	27.98 ± 1.69a	22.92–27.02	25.25 ± 1.46ab	1.00–1.22	1.11 ± 0.07a
	2018	24.43–28.90	25.86 ± 1.39a	22.19–25.87	24.16 ± 1.05b	0.98–1.17	1.07 ± 0.06a
	Mean	-	27.13 ± 1.66A	-	25.44 ± 1.67A	-	1.07 ± 0.07A
‘Polana’	2016	25.07–30.63	27.73 ± 1.74a	19.60–28.88	23.92 ± 2.25a	0.92–1.37	1.17 ± 0.12a
	2017	22.00–27.00	25.39 ± 1.52a	20.67–25.84	22.75 ± 1.68a	1.01–1.28	1.12 ± 0.10a
	2018	24.04–29.29	26.46 ± 1.58a	22.20–27.97	25.66 ± 1.44a	0.92–1.14	1.03 ± 0.07a
	Mean	-	26.53 ± 1.86A	-	24.11 ± 2.16A	-	1.11 ± 0.11A
‘Polka’	2016	25.07–30.63	27.73 ± 1.74a	19.60–28.88	23.92 ± 2.25b	0.92–1.37	1.17 ± 0.12a
	2017	23.36–29.00	26.91 ± 1.42a	25.37–28.68	26.68 ± 0.97ab	0.87–1.09	1.01 ± 0.05a
	2018	26.65–32.14	29.45 ± 1.59a	25.46–32.20	28.55 ± 1.39a	0.83–1.19	1.03 ± 0.08a
	Mean	-	28.03 ± 1.89A	-	26.38 ± 2.50A	-	1.07 ± 0.11A

Means followed by the same small letter are not significantly different within the cultivar for the years and means followed by the same capital letter do not differ between the cultivars at a significance level α = 0.05; SD standard deviation.

**Table 2:** Width of muri, distance between muri, and number of muri per unit area of exine in pollen grains of the examined *R. idaeus* cultivars.

Cultivars	Width of Muri		Distance Between Muri		Number of Muri	
					Per 10 µm^2^	
	Min.–Max.	Mean ± SD	Min.–Max.	Mean ± SD	Min.–Max.	Mean ± SD
	nm				Muri/10 µm^2^	
**Biennial Fruiting Cultivars**						
‘Glen Ample’	320–490	417. 50 ± 51.32a	560–870	786.25 ± 91.86a	5.48–9.04	8.15 ± 1.01b
‘Laszka’	240–360	298.13 ± 30.16b	490–640	574.38 ± 54.28bc	8.92–12.42	11.22 ± 1.01a
‘Radziejowa’	190–330	247.75 ± 35.87bc	660–890	781.88 ± 70.26a	7.42–13.67	10.77 ± 1.88ab
**Cultivars with Repeated Fruiting**						
‘Pokusa’	130–210	161.88 ± 23.73d	480–780	685.63 ± 90.04ab	7.67–13.56	11.55 ± 1.71a
‘Polana’	180–250	203.13 ± 24.69c	420–710	521.88 ± 69.50cd	8.03–12.67	10.98 ± 1.53a
‘Polka’	120–210	156.25 ± 29.18d	360–620	439.83 ± 68.65d	9.92–14.97	13.03 ± 1.69a

Means followed by the same small letter are not significantly different between cultivars differ at significance level α = 0.05. SD – standard deviation.

**Table 3:** Diameter of perforations and number of perforations on a fragment of colpus length and per exine unit area in pollen grains of the examined *R. idaeus* cultivars.

Cultivars	Diameter of Perforations				Number of Perforations on the Surface of			
	Min.		Max.		2-µm Long Colpus		10 µm^2^ of Exine	
	Min.–Max.	Mean ± SD	Min.–Max.	Mean±SD	Min.–Max.	Mean ± SD	Min.–Max.	Mean ± SD
	nm				Perforations/2 µm		Perforations/10 µm^2^	
**Biennial Fruiting Cultivars**								
‘Glen Ample’	90–150	117.50 ± 17.70e	100–220	151.88 ± 28.10d	3.70–7.02	5.40 ± 0.95ab	41.14–74.29	59.06 ± 10.63b
‘Laszka’	210–290	248.75 ± 22.77ab	250–450	323.75 ± 54.39a	2.08–5.18	4.24 ± 0.79b	56.66–71.02	65.01 ± 4.46b
‘Radziejowa’	200–330	258.13 ± 44,46a	210–390	306.25 ± 59.09a	4.53–8.03	6.40 ± 1.19a	70.65–130.43	91.97 ± 18.87a
**Cultivars with Repeated Fruiting**								
‘Pokusa’	120–220	167.50 ± 24.36cd	160–260	208.75 ± 31.60bc	2.01–3.97	2.68 ± 0.53c	32.84–47.06	38.47 ± 4.11c
‘Polana’	130–240	193.75 ± 38.62bc	210–310	268.75 ± 32.84ab	5.03–8.13	6.29 ± 1.01a	60.91–89.55	75.35 ± 6.71ab
‘Polka’	110–170	131.25 ± 21.25de	120–220	173.13 ± 29.38cd	5.05–8.79	6.82 ± 1.15a	49.51–88.61	71.68 ± 9.64ab

Designations as in Table 2.

**Table 4:** Characteristics of selected traits of the ectoexine in the pollen grains of the examined *R. idaeus* cultivars (nm).

Cultivars	Thickness of Tectum		Height of Columellae		Thickness of Columellae		Distance Between Columellae		Thickness of Foot Layer	
	Min.–Max.	Mean ± SD	Min.–Max.	Mean ± SD	Min.–Max.	Mean ±SD	Min.–Max.	Mean ± SD	Min.–Max.	Mean ±SD
**Biennial Fruiting Cultivars**										
‘Glen Ample’	360–540	430.75 ± 48.51a	260–510	402.50 ± 73.89a	180–310	241.88 ± 40.53a	270 ± 490	406.67 ± 69.73a	110–190	148.63 ± 23.34a
‘Laszka’	170–250	209.06 ± 23.11c	240–390	299.06 ± 49.61a	200–290	238.69 ± 25.35a	230 ± 360	296.69 ± 37.85b	70–90	77.75 ± 9.43d
‘Radziejowa’	170–280	210.88 ± 36.02bc	280–470	348.13 ± 61.03a	190–260	228.13 ± 18.34a	250 ± 380	331.88 ± 37.10b	90–120	103.13 ± 11.96bc
**Cultivars with Repeated Fruiting**										
‘Pokusa’	200–270	227.50 ± 24.90bc	270–390	335.00 ± 40.33a	170–260	208.13 ± 28.57a	260–370	321.25–37.75b	90–130	109.38 ± 9.98bc
‘Polana’	210–360	307.50 ± 43.13b	300–430	369.38 ± 54.59a	210–320	261.25 ± 35.94a	250 ± 390	339.38–39.58ab	80–130	110.04 ± 16.73c
‘Polka’	190–310	250.00 ± 43.67bc	290–440	365.01 ± 38.64a	200–320	251.88 ± 34.10a	220 ± 430	330.63–58.71b	110–150	132.50 ± 16.13a

Designations as in Table 2.

**Table 5:** Thickness of the ectoexine, endoexine, intine, and cell wall of pollen grains of *R. idaeus* cultivars (nm).

Cultivars	Ectoexine		Endoexine		Exine		Intine		Cell Wall	
	Min.–Max.	Mean ± SD	Min.–Max.	Mean ± SD	Min.–Max.	Mean ± SD	Min.–Max.	Mean ± SD	Min.–Max.	Mean ± SD
**Biennial Fruiting Cultivars**										
‘Glen Ample’	810–1090	981.88 ± 82.64a	70–110	90.42 ± 13.44ab	920–1180	1072.26 ± 82.22a	380–630	498.00 ± 74.01a	1410–1740	1570.26 ± 104.13a
‘Laszka’	500–690	585.87 ± 62.02c	50–90	76.38 ± 15.04b	580–780	662.25 ± 67.52d	250–450	316.88 ± 54.46bc	850–1177	979.13 ± 100.47b
‘Radziejowa’	550–740	662.14 ± 54.63c	60–90	82.09 ± 9.10b	620–820	744.21 ± 55.30d	320–480	393.75 ± 53.77b	880–1120	1137.96 ± 67.42b
**Cultivars with Repeated Fruiting**										
‘Pokusa’	600–740	671.88 ± 44.46bc	70–100	85.63 ± 8.92ab	690–830	757.50 ± 44.35cd	210–350	272.50 ± 37.15cd	900–1130	1030.00 ± 64.81b
‘Polana’	640–890	786.92 ± 67.50b	70–100	91.88 ± 9.11ab	740–980	878.75 ± 67.61bc	410–640	540.63 ± 66.28a	1170–1590	1419.38 ± 111.56a
‘Polka’	680–840	747.51 ± 46.98b	90–130	107.50 ± 13.42a	800–940	855.00 ± 47.61b	160–260	228.75 ± 30.30d	990–1200	1083.75 ± 55.12b

Designations as in Table 2.
